# Endoscopic obstruction is associated with higher risk of acute events requiring emergency operation in colorectal cancer patients

**DOI:** 10.1186/1749-7922-8-34

**Published:** 2013-09-08

**Authors:** Virote Chalieopanyarwong, Teeranut Boonpipattanapong, Paradee Prechawittayakul, Surasak Sangkhathat

**Affiliations:** 1 Department of Surgery, Faculty of Medicine, Prince of Songkla University, Hat Yai, Songkhla 90110, Thailand; 2 Tumor Biology Research Unit, Faculty of Medicine, Prince of Songkla University, Hat Yai, Songkhla 90110, Thailand; 3 Cancer Registry Unit, Songklanagarind Hospital, Faculty of Medicine, Prince of Songkla University, Hat Yai, Songkhla 90110, Thailand

**Keywords:** Colorectal cancers, Colonic obstruction, Surgical waiting time, Surgical outcome

## Abstract

**Introduction:**

Unplanned emergency operations in colorectal cancers (CRC) are generally associated with increased risk of operative complications. This study aimed to examine the association, if any, between an endoscopic finding of obstructing tumor and the subsequent need for an emergency operation, with the aim of determining if this finding could be useful in identifying CRC cases who are more likely to require an emergency operation.

**Methods:**

The records of CRC cases operated on in our institute during the years 2002-2011 were retrospectively reviewed regarding an endoscopic obstruction (eOB), defined as a luminal obstruction of the colon or rectum severe enough to prevent the colonoscope from passing beyond the tumor. The eOBs were analyzed against outcomes in terms of need for emergency operation, surgical complications and overall survival (OS).

**Results:**

A total of 329 CRCs which had been operated on during the study period had complete colonoscopic data. eOB was diagnosed in 209 cases (64%). Occurrence of eOB was not correlated with clinical symptoms. Colon cancer had a higher incidence of eOB (70%) than rectal cases (50%) (p-value < 0.01). eOB was significantly associated with higher tumor size and more advanced T-stage (p < 0.01). Twenty-two cases (7%) had required an emergency operation before their scheduled elective surgery. The cases with eOB had a significantly higher risk of requiring an emergency operation while waiting for their scheduled procedure (p-value < 0.01), and these emergency surgeries had more post-operative complications (36%) than elective procedures (13%) (p-value 0.01) and poorer OS (p-value < 0.01).

**Conclusion:**

Regardless of the presenting symptom, luminal obstruction severe enough to prevent further passage of a colonoscope should prompt the physician to consider an urgent surgery.

## Introduction

Colorectal cancer (CRC) is one of the common cancers in which surgery plays a crucial role in the definitive management. When a diagnosis of CRC is suspected, it is recommended by the UK National Health Service that the patient should be referred within 2 weeks [1] and treatment should be performed within one month of diagnosis [2]. However, due to resource constraints, this quick response is often impossible [3], resulting in 15-30% of CRC cases require emergency surgery due to development of acute symptoms while they await their surgery [4]. Identifying CRC patients who are likely to develop acute conditions in order to have the option of considering fast-track service could reduce problems associated with prolonged waits for necessary surgeries.

Unplanned operations in patients with colorectal cancer are associated with a higher incidence of operative complications and poorer surgical outcome than non-emergency procedures [4-6], and the most common condition that leads to emergency surgery in these patients is colonic obstruction [7]. CRC patients that are at risk of needing emergency surgery should, therefore, be prioritized. However, the clinical presentation of CRC patients is not always correlated with the severity of obstruction, this making the scheduling of prioritized surgeries a hit-and-miss decision at best. In this study, we aimed to look for a correlation between an endoscopic finding of tumor obstruction and the risk of needing emergency surgery in CRCs.

## Methods

Histologically proven colorectal adenocarcinoma patients recorded in the Cancer Registry Unit of Songklanagarind Hospital who were operated on at the institute during the period between the years 2002 and 2011 and who had a colonoscopy before their operation were included in this retrospective review. The data were retrieved from electronic medical records and reviewed regarding clinical and pathological parameters with an emphasis on the management timeline. Access and use of clinical data were approved by the Research Ethics Committee of the Faculty of Medicine, Prince of Songkla University.

Clinical management of CRC patients who were referred to our institute as an elective case usually begins with primary diagnostic confirmation by colonoscopic biopsy, followed by an appointment for an elective colectomy. Endoscopic obstruction (eOB) is diagnosed when a standard colonoscope (11.8-13.0 millimeters diameter) is unable to pass beyond the tumor. All patients were also sent for computerized tomography of their chest and abdomen as our standard pre-operative work-up while they were waiting for their surgery. During the surgical waiting period, patients who developed an emergency condition such as colonic obstruction, bleeding or tumor rupture were immediately admitted for an emergency procedure. An on-table colonic lavage technique was used in cases of left-sided colonic obstruction. Cases with an acute condition requiring immediate surgery at their initial presentation were not included in the original study. Patients who had received a prior treatment such as a colostomy from another institute or those who received neoadjuvant therapy were also excluded. In the majority of cases, laboratory tests including complete blood count, carcinoembryonic antigen and serum albumin were performed both on the first visit and on the surgical hospitalization date 4-6 weeks later. Tumor size was measured directly from the pathological specimen. Lymph node ratio (LNR) refers to the ratio between the number of positive lymph nodes and the total number of harvested nodes. A LNR cut-off of 0.35 used to determine cases with poorer prognosis in this study analysis was derived from our previous study [6]. Post-operative follow-up assessments were done through both clinical evaluation and periodic colonoscopies every 6-12 months. Adjuvant therapy was administered when indicated and the patient was physically well enough. Hospital-based follow-up data was updated until December 2012. In cases which were lost to follow-up, survival status was determined using death registry data from the regional municipal office.

Statistical analysis used Chi-squared test and logistic regression to test for any associations between eOB and the clinical parameters we were interested in. Cox’s hazard analysis was used to study association between eOB and emergency surgery. Survival outcome was analyzed in terms of overall survival (OS). Log-rank test and Kaplan-Meier survival analysis were used for survival comparison. Data are presented as hazard ratios (HR) with a 95% confidence interval (95% CI), with p-values of less than 0.05 considered statistically significant.

## Results

### Patients data

A total of 329 consecutive cases (191 males and 138 females) who were operated on during the study period and had complete data concerning colonoscopic findings were included in the analysis. Their mean age was 62 years with 193 patients (59%) aged more than 60 years. The primary tumor was in the rectum in 94 cases (29%), the colon in 223 cases (68%) and multiple sites in 12 cases (4%). The most common presenting symptoms were abdominal pain (29%), bowel habit change (26%) and lower gastrointestinal bleeding (26%). Decreased stool frequency was the predominating symptom in 19 cases (6%). Other pathological parameters and their association with survival are presented in Table1. The average waiting time from the first hospital visit to the operation was 35 days.

**Table 1:** Selected demographic and medical parameters and their association with 5-year overall survival (OS) and modes of surgery

		**Survival probability**		**Emergency surgery**	
**Parameter**	**No. (cases) (%)**	**5-year OS (%)**	**Log-rank p-value**	**(cases) (%)**	**p-value**
All	329	64.1	-	22 (7)	-
Sex			0.5		0.73
male	191 (58)	62.4		12 (6)	
female	138 (42)	66.5		10 (7)	
Age			0.51		0.35
< 60 years	136 (41)	66.7		7 (5)	
≥ 60 years	193 (59)	62.3		15 (8)	
Co-morbidity			0.71		0.97
Absent	193 (59)	65.5		13 (7)	
Present	136 (41)	61.7		9 (7)	
Serum CEA			< 0.01		0.32
< 5 ng/ml	144 (59)	71.1		8 (6)	
≥ 5 ng/ml	102 (41)	54.8		9 (9)	
Tumor site			0.32		0.79
Rectum	94 (29)	56.8		5 (5)	
Colon	223 (68)	66.8		16 (7)	
T			0.02		0.18
T0-2	47 (14)	75.9		1 (2)	
T3-4	282 (86)	62		22 (8)	
N			< 0.01		0.34
N0	171 (53)	78.7		9 (5)	
N1-2	152 (47)	49.4		12 (8)	
M			< 0.01		0.02
M0	281 (85)	72.1		15 (5)	
M1	48 (15)	18.5		7 (15)	
Tumor differentiation			0.16		0.77
Well/Moderate	279 (92)	64.9		18 (7)	
Poor	25 (8)	58.6		2 (8)	
Lymphovascular invasion			< 0.01		0.12
Absent	276 (84)	69		16 (6)	
Present	51 (16)	35.3		6 (12)	
Lymph node ratio			< 0.01		0.53
< 0.35	273 (86)	72.7		17 (6)	
≥ 0.35	46 (14)	23.6		4 (9)	
Endoscopic obstruction			0.73		< 0.01
Absent	120 (37)	67.2		2(2)	
Present	209 (64)	62.3		20 (10)	
Mode of operation			< 0.01		-
Elective	307 (93)	66.4		-	
Emergency	22 (7)	32.3		-	

*CEA* carcinoembryonic antigen.

### Endoscopic obstruction and factors associated with this finding

On colonoscopy, the endoscope could not be passed beyond the tumor mass in 209 cases (63%). Clinical symptoms suggestive of early obstruction including decreased stool frequency or change in bowel habit were not significantly correlated with eOB (p-values 0.64 and 0.45, respectively). Although a primary tumor situated at the right colon had a significantly lower incidence of predominating obstructive symptoms (1%) than a left-sided CRC (8%) (p-value 0.02), the right-sided tumors had a higher incidence of eOB (72%) when compared to those on the left (60%, p-value 0.047). Colonic tumors had a higher incidence of eOB (70%) than rectal tumors (50%) (p-value < 0.01).

Considering tumor size, CRC with eOB had a significantly larger size (5.9 cm compared with 5.2 cm, p-value < 0.01) and a higher frequency of T3-4 lesions (91% compared to 75%, p-value < 0.01). Also, eOBs were associated with lower serum albumin level (3.7 g/dl, compared to 3.9 g/dl, p-value 0.04) and lower hemoglobin level (10.5 g/dl, compared to 11.2 g/dl, p-value < 0.01) (Table 2).

**Table 2:** Association between selected clinicopathological parameters and endoscopic obstruction

**Parameter**	**No. (cases) (%)**	**Endoscopic obstruction (%)**	**p-value**
All	329	120 (37)	-
Tumor site			< 0.01
Rectum	94 (29)	47 (50)	
Colon	223 (68)	155 (70)	
Tumor side			0.047
Left colon and rectum	224 (68)	135 (60)	
Right colon	93 (28)	67 (72)	
serum CEA			0.31
< 5 ng/ml	144 (59)	87 (60)	
≥ 5 ng/ml	102 (41)	68 (67)	
Tumor size			< 0.01
< 5.5 cm	181 (57)	104 (57)	
≥ 5.5 cm	136 (43)	98 (72)	
T			< 0.01
T0-2	47 (14)	18 (38)	
T3-4	282 (86)	191 (68)	
N			0.90
N0	171 (53)	108 (63)	
N1-2	152 (47)	97 (64)	
M			0.07
M0	281 (85)	173 (61)	
M1	48 (15)	36 (75)	
Tumor differentiation			0.63
Well/Moderate	279 (92)	181 (64)	
Poor	25 (8)	15 (60)	
Lymphovascular invasion			0.18
Absent	276 (84)	179 (64)	
Present	51 (16)	28 (59)	

*CEA* carcinoembryonic antigen.

### Significance of endoscopic obstruction on mode of operation and outcome

**Figure 1:**
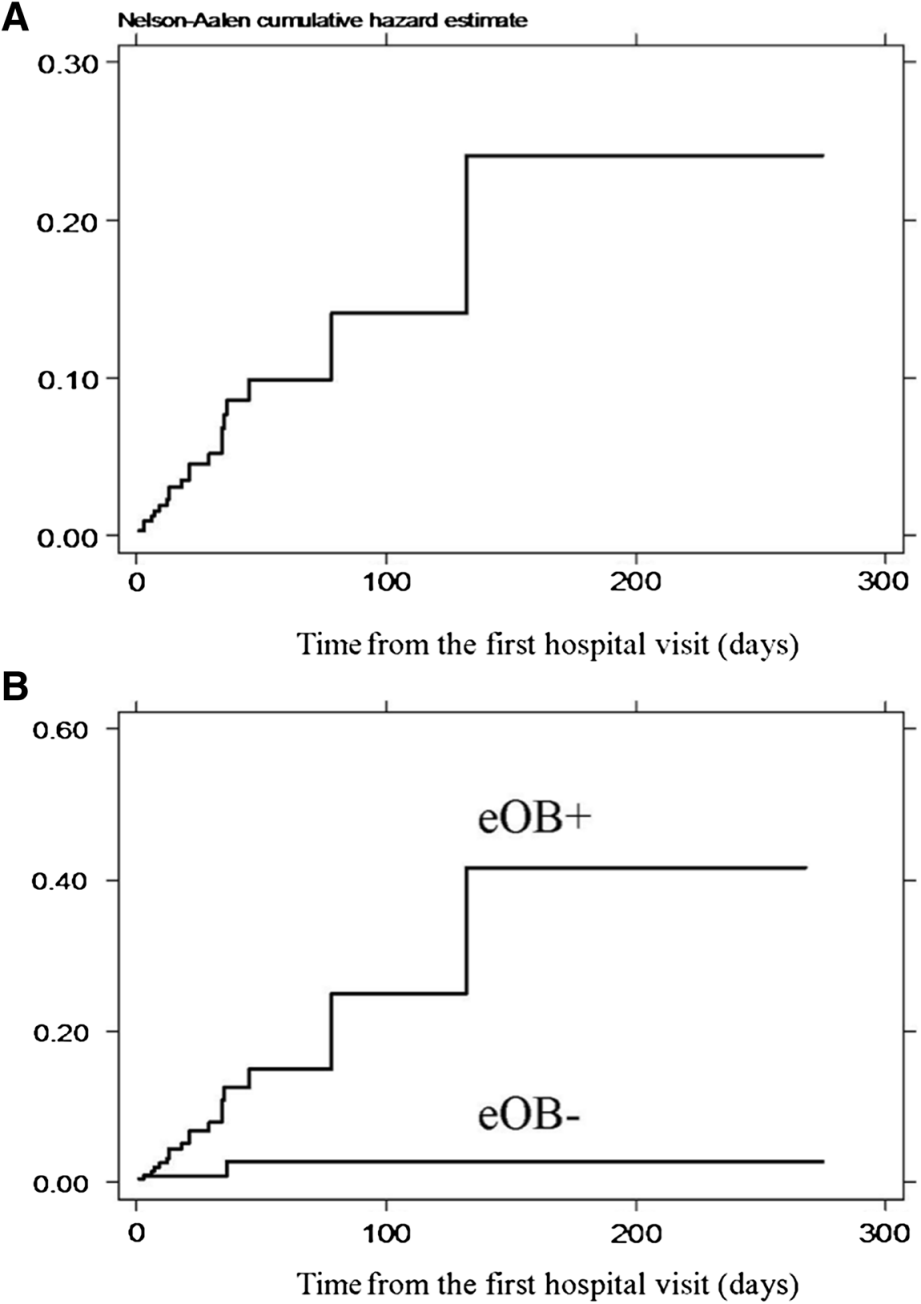
Probability of requiring an emergency operation A: overall B: comparing between cases with and without endoscopic obstruction.

Twenty-two cases (7%) required an emergency operation before their scheduled elective procedure. The emergency surgery requirement was significantly higher in eOB cases (10%), compared to those without obstruction (2%). Cases with an eOB had a significantly higher chance of requiring an emergency operation at a Cox’s hazard ratio of 6.9 (95% confidence interval 1.6-29.7). Among cases with eOB, the frequency of cases requiring emergency surgery was not significantly different between rectal cases (9%) and colonic cases (10%) (p-value 0.8). The median time from colonoscopy to operation in the emergency cases was 14 days. The cumulative incidences of emergency surgery in all cases at 15, 30 and 60 days of surgical waiting were 3%, 5% and 9%, respectively (Figure 1). The 60-day cumulative emergency operation rate was 14% in those with an obstructing tumor, compared to 3% in cases in which an endoscope could be passed beyond the tumor (p-value < 0.01). The reasons for the emergency surgery included complete colonic obstruction presenting as abdominal pain, vomiting and obstipation in 20 cases and 1 case each of gastrointestinal bleeding and tumor perforation. The emergency procedure was a definitive colorectal resection in all 22 cases. Patients who underwent emergency surgery had a higher incidence of distant metastasis (32% compared to 13% in elective cases, p-value 0.02).

Operative complications occurred in 48 cases (15%). Patients who underwent an emergency operation had a higher rate of post-operative complications (36%) than those who had surgery according to their elective schedule (13%, p-value < 0.01). (Table 3) On survival analysis, although eOB was not directly associated with overall survival, requiring emergency operation had a statistically significant impact on poorer overall survival (p-value < 0.01).

**Table 3:** Post-operative complications according to mode of surgery (some cases had more than one complication)

	**Emergency (N:22)**	**Elective (N:307)**
Wound infection	5 (23%)	13 (4%)
Wound dehiscence/evisceration	2 (9%)	5 (2%)
Anastomotic leakage/fistula	2 (9%)	10 (3%)
Intestinal obstruction	0 (0%)	5 (2%)
Other (collection, seroma, etc.)	1 (5%)	10 (3%)

## Discussion

Previous studies have suggested that CRCs that present with acute symptoms and require emergency surgery have more aggressive behavior and higher tumor stages [4,8]. Consistent with those findings, our study found that CRC patients who underwent emergency surgery had a more advanced stage tumor, which may partly explain the poorer survival. In addition, unplanned emergency operations are inferior to elective surgeries in terms of inadequate control of any underlying co-morbidities. For these reasons, it could be expected that procedures done in an emergency setting post a higher risk of operative complications. Obstruction and perforation are common problems that bring CRC patients to an emergency surgery before their scheduled surgery [5,7,9]. The number of emergency surgeries in our series was relatively lower than other previous reports [4,5,7-9], which might be explained by the fact that we did not include cases who first presented with emergency conditions in our analysis.

Providing fast-track service for these higher risk CRC patients may help in reducing acute events that require emergency surgery and its related higher morbidity [10]. Our study found that clinical symptoms alone were not adequate in determining such high-risk patients, especially when the tumor was situated on the right colon. The pre-operative colonoscopy is an objective study that should be performed in all cases suspected of CRC, as in addition to a tissue biopsy for histological confirmation of malignancy, severity of luminal obstruction can be evaluated. We also found that a luminal obstruction was associated with larger tumor size and T-stage, but not histological grade. Moreover, eOB was also correlated with poorer nutritional status in our cases, as evidenced by lower serum albumin and hemoglobin. Above all, the evidence of eOB was associated with required emergency surgery. Overall, the data from our study suggest that patients with eOB should be reevaluated carefully and considered for fast-track urgent surgery. The average surgical waiting time in the study CRC cases was 35 days. If all of our cases are considered as on the same elective list, 10% of cases with eOB and 2% of non-eOB cases required an emergency operation. However, if the patients with eOB had been scheduled for surgery within 2 weeks of their first hospital visit, the overall number of emergency surgeries would have been reduced to 5%.

Use of a self-expandible metallic stent as a bridge-to-surgery method has been recently proposed, not only as a time-buying strategy, but also to allow for more adequate pre-operative staging and bowel preparation [11]. The stent procedure has one notable technical limitation, however, in that it can be applied only for an obstruction in the left colon and rectum. Although various retrospective case series have reported the benefits of this intervention [12-14], there are yet no good quality data to support its clinical advantage over emergency surgery.

In conclusion, our study found that a luminal obstruction detected by endoscopy was significantly associated with locally advanced tumor. This group of CRC patients had a higher risk of requiring an unplanned operation. The data suggest that this endoscopic finding should be regarded as an indication that these patients should be considered for fast-track surgical scheduling list.

## Competing interests

The authors declare that they have no competing interests.

## Authors’ contribution

VC initiated the research project and collected all data. TB provided clinical data and reviewed the quality of data collection. PP provided community based follow-up data. SS managed the project, analyzed data and prepared the manuscript draft. All authors read and approved the final manuscript.

## References

[B1] Department of Health The NHS Cancer Plan 2000 London: Department of Health

[B2] Duff SE Wood C McCredie V Levine E Saunders MP O’Dwyer ST Waiting times for treatment of rectal cancer in North West England J R Soc Med 2004 8 117 118 10.1258/jrsm.97.3.117 14996956 PMC1079319

[B3] Hanna SJ Muneer A Khalil KH The 2-week wait for suspected cancer: time for a rethink? Int J Clin Pract 2005 8 1334 1339 10.1111/j.1368-5031.2005.00687.x 16236089

[B4] Wong SK Jalaludin BB Morgan MJ Berthelsen AS Morgan A Gatenby AH Fulham SB Tumor pathology and long-term survival in emergency colorectal cancer Dis Colon Rectum 2008 8 223 230 10.1007/s10350-007-9094-2 18097722

[B5] Bass G Fleming C Conneely J Martin Z Mealy K Emergency first presentation of colorectal cancer predicts significantly poorer outcomes: a review of 356 consecutive Irish patients Dis Colon Rectum 2009 8 678 684 10.1007/DCR.0b013e3181a1d8c9 19404074

[B6] Kritsanasakul A Boonpipattanapong T Wanitsuwan W Phukaoloun M Prechawittayakul P Sangkhathat S Impact of lymph node retrieval on surgical outcomes in colorectal cancers J Surg Oncol 2012 8 238 242 10.1002/jso.22156 22886537

[B7] Cuffy M Abir F Audisio RA Longo WE Colorectal cancer presenting as surgical emergencies Surg Oncol 2004 8 149 157 10.1016/j.suronc.2004.08.002 15572097

[B8] Ghazi S Berg E Lindblom A Lindforss U Low-Risk Colorectal Cancer Study Group Clinicopathological analysis of colorectal cancer: a comparison between emergency and elective surgical cases World J Surg Oncol 2013 8 133 10.1186/1477-7819-11-133 23758762 PMC3687686

[B9] Chen HS Sheen-Chen SM Obstruction and perforation in colorectal adenocarcinoma: an analysis of prognosis and current trends Surgery 2000 8 370 376 10.1067/msy.2000.104674 10776426

[B10] Scott MA Knight A Brown K Novell JR A single common urgent pathway for all colorectal referrals reduces time to diagnosis and treatment Colorectal Dis 2006 8 766 771 10.1111/j.1463-1318.2006.01034.x 17032322

[B11] Baik SH Kim NK Cho HW Lee KY Sohn SK Cho CH Kim TI Kim WH Clinical outcomes of metallic stent insertion for obstructive colorectal cancer Hepatogastroenterol 2006 8 183 187 16608020

[B12] Ng KC Law WL Lee YM Choi HK Seto CL Ho JW Self-expanding metallic stent as a bridge to surgery versus emergency resection for obstructing left-sided colorectal cancer: a case-matched study J Gastrointest Surg 2006 8 798 803 10.1016/j.gassur.2006.02.006 16769535

[B13] van Hooft JE Bemelman WA Oldenburg B Marinelli AW Holzik MF Grubben MJ Sprangers MA Dijkgraaf MG Fockens P collaborative Dutch Stent-in study group Colonic stenting versus emergency surgery for acute left-sided malignant colonic obstruction: a multicentre randomised trial Lancet Oncol 2011 8 344 352 10.1016/S1470-2045(11)70035-3 21398178

[B14] Zhang Y Shi J Shi B Song CY Xie WF Chen YX Self-expanding metallic stent as a bridge to surgery versus emergency surgery for obstructive colorectal cancer: a meta-analysis Surg Endosc 2012 8 110 119 10.1007/s00464-011-1835-6 21789642

